# Investigation of glycaemic traits in psychiatric disorders using Mendelian randomisation revealed a causal relationship with anorexia nervosa

**DOI:** 10.1038/s41386-020-00847-w

**Published:** 2020-09-13

**Authors:** Danielle M. Adams, William R. Reay, Michael P. Geaghan, Murray J. Cairns

**Affiliations:** 1 School of Biomedical Sciences and Pharmacy, The University of Newcastle, Callaghan, NSW Australia; 2 Centre for Brain and Mental Health Research, Hunter Medical Research Institute, Newcastle, NSW Australia

**Keywords:** Genetics, Risk factors, Psychiatric disorders

## Abstract

Data from observational studies have suggested an involvement of abnormal glycaemic regulation in the pathophysiology of psychiatric illness. This may be an attractive target for clinical intervention as glycaemia can be modulated by both lifestyle factors and pharmacological agents. However, observational studies are inherently confounded, and therefore, causal relationships cannot be reliably established. We employed genetic variants rigorously associated with three glycaemic traits (fasting glucose, fasting insulin, and glycated haemoglobin) as instrumental variables in a two-sample Mendelian randomisation analysis to investigate the causal effect of these measures on the risk for eight psychiatric disorders. A significant protective effect of a natural log transformed pmol/L increase in fasting insulin levels was observed for anorexia nervosa after the application of multiple testing correction (OR = 0.48 [95% CI: 0.33-0.71]—inverse-variance weighted estimate). There was no consistently strong evidence for a causal effect of glycaemic factors on the other seven psychiatric disorders considered. The relationship between fasting insulin and anorexia nervosa was supported by a suite of sensitivity analyses, with no statistical evidence of instrument heterogeneity or horizontal pleiotropy. Further investigation is required to explore the relationship between insulin levels and anorexia.

## Introduction

Psychiatric disorders are complex phenotypes aetiologically influenced by a range of environmental [1, 2] and genetic factors [3–7]. Currently, psychiatric disorders are treated with a combination of medication [8, 9] and psychotherapy approaches [10]. Often these interventions address the symptoms of the disease without targeting the underlying mechanisms of action, and thus, managing psychiatric disorders remains difficult for many patients [11, 12]. To address this, we need to better understand the risk factors and underlying pathophysiology of these conditions such that novel intervention strategies can be implemented.

There has been increasing interest in the relationship between glycaemic regulation and psychiatric illness. Dysglycaemia has well characterised systemic effects, however, its importance in the brain is often underappreciated. Insulin has been implicated in many neurological processes including synaptic plasticity and cognition [13–15], whilst neurons are dependent on glucose as their major energy source [16]. A disproportionately high burden of comorbid type 2 diabetes has been observed in several psychiatric disorders, including: schizophrenia (SZ) [17], bipolar disorder (BP) [17, 18], major depressive disorder (MDD) [17], autism spectrum disorder (ASD) [19], and Tourette’s syndrome (TS) [20]. In addition, glycaemic abnormalities have been observed through direct serum measurement, including an association of elevated glycated haemoglobin with attention deficit hyperactive disorder (ADHD) [21], insulin resistance with psychotic experiences [22], and elevated insulin sensitivity in anorexia [23]. Lifestyle factors and metabolic consequences of antipsychotic medication, such as weight gain [24, 25], likely contribute to these associations. However, data from treatment naïve, first episode psychosis patients provides evidence of glycaemic dysregulation in these disorders beyond what is directly attributable to medication effects and lifestyle [22, 26, 27]. This relationship is further supported by genetic studies. For instance, linkage disequilibrium score regression (LDSC) has demonstrated a negative genetic correlation between anorexia and both fasting inulin and glucose as indexed by common genomic variation [28]. Polygenic risk score for schizophrenia has also been associated with insulin resistance [29], whilst there is evidence of shared genome-wide association study (GWAS) association signals for schizophrenia and type 2 diabetes which display statistical colocalisation [30]. Given the importance of glycaemic regulation in the brain, and the direct significance of insulin signalling, these data suggest that dysglycaemia may be involved in the pathogenesis of psychiatric disorders. This could have implications for clinical monitoring and precision medicine as this system can be modulated through direct pharmacological intervention and lifestyle alterations.

The literature supporting the relationship between glycaemic traits and psychiatric disorders is largely composed of observational studies, preventing direct causal inferences. Randomised controlled trials (RCT) are viewed as an effective method to overcome this, however they are expensive and difficult to conduct with large sample sizes. An alternative method for inferring causal relationships between traits is Mendelian randomisation (MR), which is an analytical method to determine the causal effect of an exposure on an outcome by comparing the association of genetic instrumental variables (IV) with the outcome, relative to the IV effect on the exposure [31]. Genetic variants which are rigorously associated with the exposure — discovered through GWAS — are selected as IVs, which in turn serve as proxies for the exposure. Two-sample MR is particularly advantageous as only GWAS summary statistics are required for the exposure and outcome traits of interest. Mendel’s principle of independent assortment and random segregation ensure that these IVs will be randomized, and thus, their random distribution in the population emulates the random distribution of an exposure for individuals in an RCT [32, 33]. In the present study, we have applied this approach to probe the causal effects of glycaemic traits on the risk for psychiatric disorders and observed a significant protective effect of elevated fasting insulin levels on the risk of anorexia nervosa (AN).

## Methods

### Selection of genetic instrumental variables

IVs for MR are genetic variants associated with a particular effect size for a trait. There are three main assumptions which underlie the use of these IVs: [34–36]

IV1: the variant is rigorously associated with the exposure;

IV2: the variant is independent of all confounders of the exposure-outcome relationship (“exclusion-restriction assumption”); and

IV3: the variant is associated with the outcome only by acting through the exposure (independent conditional on the exposure and confounders).

IV1 is the only assumption which can be directly quantified [37]; thus, we implement models (described below) to evaluate evidence for violations of these core assumptions. Specifically, pleiotropy, wherein a variant is associated with multiple phenotypes, may invalidate an IV if said pleiotropy constitutes an alternate causal pathway between the variant and the outcome (horizontal pleiotropy) [32].

We chose three core glycaemic traits to use as exposures in this study for which well-powered GWAS data were available: fasting insulin, fasting glucose, and glycated haemoglobin (HbA1c). IVs were genome-wide significant SNPs (*P* < 5 × 10^−8^, such that IV1 is satisfied) from the largest GWAS available for each trait [38, 39]. Fasting insulin and fasting glucose data were obtained from the same meta-analysis of non-diabetic individuals of European ancestry (fasting insulin: *N* = 108,557, unit of effect = *ln* pmol/L; fasting glucose: *N* = 133,310, unit of effect = mmol/L). Fasting insulin GWAS data were originally obtained from serum samples. The fasting insulin GWAS provided summary statistics with and without covariation for body mass index (BMI). Both SNP effect sizes were considered as IVs due to the complex relationship between insulin and weight gain [40–42]. Fasting glucose data were obtained from either plasma or from whole blood and corrected to plasma levels [38]. IVs for HbA1c were obtained from the European subset of a GWAS meta-analysis (*N* = 123,665, unit of effect = % HbA1c). Genome-wide significant SNPs were further categorised in this study as those acting through glycaemic pathways and those acting through erythrocytic pathways via annotation with GWAS catalogue associations as described in Wheeler et al. [39]. We chose to utilise the full set of significant SNPs as IVs, as well as the subset of the lead SNPs specifically annotated as glycaemic, to reduce potential horizontal pleiotropy (gHbAlc). The *F* statistic was calculated using equation one where *R*^*2*^ is the variance in the outcome explained by each SNP (estimated using the squared sum of the get_r_from_pn() function), *k* is the number of viable IVs and *N* is the sample size (Table 1), demonstrating all IVs were sufficiently strong (*F* > 10).

1 $$F = \frac{{R^2(N - k - 1)}}{{\left( {1 - R^2} \right)k}}.$$

**Table 1:** Instrumental variables selected for each glycaemic exposure.

Exposure	Number of IVs	Variance explained (%)	F statistic	Sample size	Units
Fasting insulin	14	0.64	40.66	108,557	ln pmol/L
Fasting glucose	32	3.31	130.27	133,310	mmol/L
Fasting glycated haemoglobin (all)	38	2.42	80.65	123,665	% glycated haemoglobin
Fasting glycated haemoglobin (glycaemic)	15	0.85	70.39	123,665	% glycated haemoglobin

The number of IVs, variance explained, *F* statistic, sample size and units are described for the glycaemic exposures. The *F* statistic was calculated from the number of IVs, variance explained and sample size as described previously [90]. The variance explained was only from the IVs utilised in this study [43].

Hereafter, we refer to four exposures, as opposed to three, as there are two IV sets used for HbA1c, all SNPs and SNPs annotated as glycaemic (gHbA1c). IVs for all four exposures (fasting insulin, fasting glucose, HbA1c, and gHbA1c) were clumped to remove variants in linkage disequilibrium (*r*^2^ < 0.001) upon importation into the TwoSampleMR (version 0.4.25) R package [43].

### Outcome data

We selected eight psychiatric disorders for which GWAS data were available as the outcome traits in this study: AN, ADHD, ASD, BP, MDD, obsessive compulsive disorder (OCD), SZ, and TS. Outcome data were restricted to GWAS summary statistics from subjects of European ancestry in accordance with the exposure data, with the respective sample sizes as follows – AN: *N* = 72,515 [28], ADHD: *N* = 53,293 [European subset] [44], ASD: *N* = 46,351 [45], BP: *N* = 51,710 [46], MDD: *N* = 1,730,005 [23andMe cohorts were not included in public release of the summary statistics from the psychiatric genomics consortium] [47], OCD: *N* = 9725 [48], SZ: *N* = 105,318 [49], and TS: *N* = 14,307 [50]. The collection of individual demographic, clinical and genetic data was supervised by the respective institutional ethics review boards for each study after obtaining informed consent of participants. No further ethics approval was required for our analyses as we only accessed summary level meta-data. The number of genome-wide significant SNPs and SNP-based heritability for the outcome GWAS are detailed in Table 2. There was no sample overlap between the glycaemic exposures and the psychiatric outcomes to the best of our knowledge based on the contributing cohorts for each GWAS. However, this cannot be proven definitely, as we do not have access to the raw genotype/phenotype data of each of the studies. We also performed MR in the opposite direction using genome-wide significant variants for each of the above psychiatric traits as IVs. In this instance, we utilised a different GWAS of fasting insulin and FG as our outcome. The fasting insulin summary statistics produced by Scott et al. used as MR IVs followed up ~66,000 SNPs from previous GWAS. Whilst this is the largest sample size GWAS for this trait, the limited number of SNPs made it unsuitable to identify IV-outcome effects. Therefore, we utilised the smaller sample size GWAS summary statistics from Manning et al. (*N* = 33,823) with more SNPs available as a greater number of psychiatric IVs were encompassed by these summary statistics.

**Table 2:** Characteristics of the psychiatric genome-wide association studies utilised as outcomes.

Outcome	Cases/Controls	SNP heritability	GWAS hits
Attention deficit/ hyperactivity disorder	19,099/34,194	0.22	12
Anorexia nervosa	16,992/55,525	0.11	8
Autism spectrum disorder	18,381/27,969	0.12	5
Bipolar disorder	20,352/31,358	0.17	19
Major depressive disorder	59,851/113,154	0.08 (0.10)^a^	44 (5)^b^
Obsessive compulsive disorder	2688/7037	0.28	0
Schizophrenia	40,675/64,643	0.23	145
Tourette’s syndrome	4819/9488	0.21	1

SNP heritability reported on the liability scale assuming the following population prevalence: attention deficit hyperactive disorder = 5%, anorexia = 0.9%, autism = 1.2%, bipolar = 0.5%, major depressive disorder = 15%, schizophrenia = 0.7%, obsessive compulsive disorder = 2.5%, Tourette’s syndrome = 0.8%. GWAS hits denotes the number of independent, genome-wide significant variants reported by the original study.

^a^The reported heritability estimate by the MDD publication is given, with the liability scale SNP-heritability for the publicly available subset in parentheses.

^b^The MDD GWAS study reports 44 genome-wide significant SNPs, however, only a subset of this cohort has publicly available summary stats, and this subset has 5 genome-wide significant SNPs.

### Two sample Mendelian randomisation approach

First, we investigated the effect of fasting insulin, fasting glucose, HbA1c, and gHbA1c on the risk for each of the eight psychiatric disorders described above using an inverse-variance weighted effect model with multiplicative random effects (IVW) [51]. The positive strand was inferred where possible otherwise palindromic SNPs were removed [52]. We performed composite approaches and sensitivity analyses for exposure-outcome relationships which were significant after Bonferroni correction for the four exposures tested for eight outcomes [*P* < 1.56 × 10^−3^, *α* = 0.05/(8 × 4)]. The reverse MR analyses utilised the same IVW estimator approach. Beta estimates for nominally significant models with a binary exposure were converted to the liability scale assuming a population prevalence of 0.7% and 0.9% for SZ and AN, respectively [53].

The IVW model is limited such that even one invalid IV can bias the overall estimate. Therefore, for estimates with corrected significance we sought to overcome this limitation by using the outlier-robust MR-Pleiotropy Residual Sum and Outlier (MR-PRESSO) method [51, 54]. MR-PRESSO is underpinned by the residual sum of squares (RSS), which serves as a heterogeneity measure of ratio estimates. Specifically, an IVW estimate using the IVs is calculated in a leave-one out fashion; if the RSS is decreased significantly relative to a simulated Gaussian distribution of expected RSS, then that variant is excluded from the IVW model. Simulations have demonstrated that this methodology is best suited to instances when less than half of the IVs exhibit horizontal pleiotropy [54]. Three additional MR approaches were implemented to better account for potential invalid IVs: a weighted median estimate, a weighted mode estimate, and MR-Egger. The weighted median model takes the median of the ratio estimates (as opposed to the mean in the IVW model), such that upweighting (with second order weights [55]) is applied to ratio estimates with greater precision [36]. An advantage of this approach is that it is subject to the ‘majority valid’ assumption, whereby an unbiased causal estimate will still be obtained if less than 50% of the model weighting arises from invalid IVs. Mode-based estimators are subject to the related ‘plurality valid’ assumption [56]. Finally, an MR-Egger model was constructed [35]. This is an adaption of Egger regression wherein the exposure effect is regressed against the outcome with an intercept term added to represent the average pleiotropic effect. The *I*^2^ statistic for the IV-exposure effects was calculated to assess the relative strength of the no-measurement error assumption, and thus, the suitability of using an MR-Egger model. The conventional threshold of *I*^2^ > 0.9 was utilised to deem the IVs appropriate for MR-Egger [57]. We also estimated the effect of fasting insulin on anorexia using generalised summary-data-based Mendelian Randomisation (GSMR) [58]. GSMR is statistically similar to an IVW approach but is implemented using an R package from an independent research group, which serves as an important replication from a technical perspective.

### Sensitivity and pleiotropy analyses

The key assumption of the MR-Egger model is referred to as Instrument Strength Independent of Direct Effect (InSIDE), which assumes that there is no significant correlation between direct IV effects on the outcome and genetic association of IVs with the exposure [35, 59]. In other words, the InSIDE assumption is violated if pleiotropic effects act through a confounder of the exposure-outcome association. We employed three primary methods to evaluate evidence for unbalanced pleiotropy: The intercept from the MR Egger model, Cochran’s *Q*, and the MR PRESSO global pleiotropy test. First, the Egger intercept was tested as to whether it was significantly different from zero, as a nonzero intercept may indicate unbalanced pleiotropy or violation of the InSIDE assumption, given that the intercept represents the mean pleiotropic effect [60]. Furthermore, as heterogeneity amongst the IV exposure-outcome ratio estimates could be caused by horizontal pleiotropy, we quantified this heterogeneity using Cochran’s Q statistic [43, 61, 62]. Finally, a global pleiotropy test was implemented via the MR-PRESSO framework, which utilised the expected and observed RSS [54]. A leave-one-out analysis was then performed to assess whether causal estimates are biased by a single IV, which may indicate the presence of outliers, and the sensitivity of the estimate to said outliers [43]. The MR Steiger directionality test utilizes the phenotypic variance explained by IV SNPs, comparing the instruments’ association with the exposure and outcome to determine if there is evidence that the assumed direction of causality is correct [63]. For binary traits, the trait population prevalence was used to calculate variance explained and convert to the liability scale using the lower and upper bounds of population prevalence estimates used by the GWAS for consistency (0.9% and 4% respectively for anorexia) [28, 64, 65]. To investigate the significance of BMI-associated SNPs [66] on the relationship between outcome and exposure, SNPs associated with both traits were removed and the IVW estimate recalculated. All MR analyses were performed using the TwoSampleMR v0.4.25 package [43] in R v3.6.1 [67], with the exception of the MR-PRESSO model which utilised the MRPRESSO package v1.0 and the GSMR estimate performed with the gsmr package v1.0.9.

### Latent causal variable model to estimate the genetic causality proportion of fasting insulin on risk for anorexia nervosa

Fasting insulin and AN display significant genome-wide genomic correlation as indexed by LDSC [28]. This correlation may confound MR, and thus, we implemented a latent causal variable model (LCV) as an additional approach to investigate whether this correlation represents a causal relationship [68]. Briefly, the LCV method assumes that a latent variable mediates the genetic correlation between two traits and tests whether this latent variable displays stronger correlation with either of the traits. Using fourth moments of the bivariate effect size distributions of all SNPs in both GWAS datasets, and their LD structure, a posterior mean estimate of the genetic causality proportion (GCP) is derived which quantifies how much of the genomic architecture of one trait effects another. GCP values range from −1 to 1, with more positive values indicating greater partial genetic causality of trait one on two, and vice versa for more negative values. Full genetic causality is described as GCP = 1 or −1, which is rare in practice [68], with partial genetic causality occurring within these limits. A two-sided *t* test was used to assess whether the estimated GCP was significantly different from zero. The RunLCV.R and MomentFunctions.R scripts were leveraged to perform these analyses (https://github.com/lukejoconnor/LCV/tree/master/R). The Manning et al. fasting insulin GWAS was once more utilised for these analyses due to its more complete summary statistics and because it was the basis for the previously performed LDSC between anorexia and fasting insulin [28, 69]. Both summary statistics were cleaned and formatted in a standardised way (“munged”) prior to analysis with the LCV model [68, 70, 71].

## Results

### Evidence of a protective effect of fasting insulin on anorexia nervosa

**Fig. 1:**
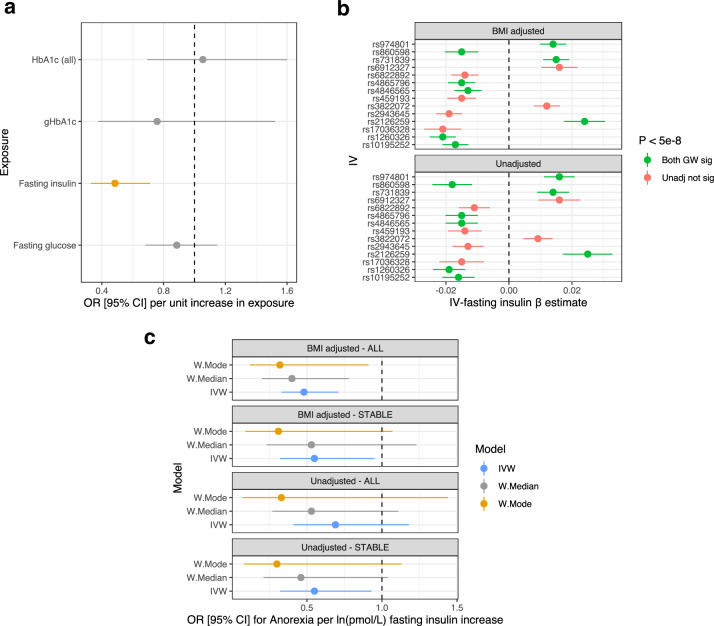
Effect of glycaemic traits on the risk of anorexia nervosa. **a** Forest plot of the IVW estimates of the relationship between glycaemic exposures and anorexia nervosa. The estimates represent an odds ratio (OR) per unit increase in the exposure, with the error bars denoting the 95% confidence interval. The glycaemic exposures were as follows: fasting insulin, fasting glucose. glycated haemoglobin (HbA1c (all)), and a subset of glycaemic glycaeted haemoglobin lead SNPs. There was a significant protective effect of fasting insulin on anorexia nervosa after the application of multiple testing correction, and thus, that estimate is shaded orange. **b** Comparison of the IV-exposure association effect size for fasting insulin instrumental variables with, and without, phenotypic covariation for body mass index (BMI). The two panels plot the beta estimate of the 14 SNP-fasting insulin associations (error bars are 95% confidence interval) derived from the GWAS with or without adjustment for BMI. IV-estimates highlighted green were associated with fasting insulin at genome-wide significance (*P* < 5 × 10^−8^) irrespective of BMI adjustment (“both GW sig”), whilst red shaded SNP-exposure effects were only significant upon covariation for BMI. **c** Sensitivity analyses of BMI adjusted and unadjusted fasting insulin instrumental variables. We defined the instrumental variables for fasting insulin as follows: all IVs unadjusted for BMI, all IVs adjusted for BMI, IVs significant irrespective of BMI (stable IVs – estimates with and without BMI adjustment used). The forest plot denotes three MR estimators (IVW, weighted median, and weighted mode) using each of these IV subsets; each point represents the odds ratio for anorexia nervosa per natural log transformed pmol/L fasting insulin.

The selected glycaemic IVs explained ~3.33%, 0.64%, 2.42%, 0.85% of exposure variance of fasting glucose, fasting insulin, and glycated haemoglobin levels (HbA1c, gHbA1c), respectively. IVs were selected by clumping for LD to remove correlated variants and excluding palindromes for which the correct strand could not be inferred. An IVW model was used to estimate the casual effect of the four exposures on the eight psychiatric disorder outcomes. We revealed a significant protective effect of a unit increase [*ln*(pmol/L)] in fasting insulin levels on anorexia after the applying Bonferroni correction (OR = 0.48, [95% CI:0.33–0.71], *P* = 2.27 × 10^−4^) (Fig. 1a). It should be noted that this model utilised fasting insulin IVs from a GWAS adjusted for BMI, IVs unadjusted for BMI attenuated the causal estimate, although the point estimate was directionally consistent: OR = 0.69 [95% CI: 0.41–1.18], *P* = 0.178. The impact of covariation for BMI is discussed further in the subsequent section. In addition, the relationship between fasting insulin and MDD was nominally significant (uncorrected *P* < 0.05) [OR = 0.85, 95% CI: 0.74–0.97, *P* = 0.015], whilst all other causal estimates were not significant after utilising either the Bonferroni or less conservative Benjamini-Hochberg method for multiple testing correction. (Supplementary Table 1)*.*

**Fig. 2:**
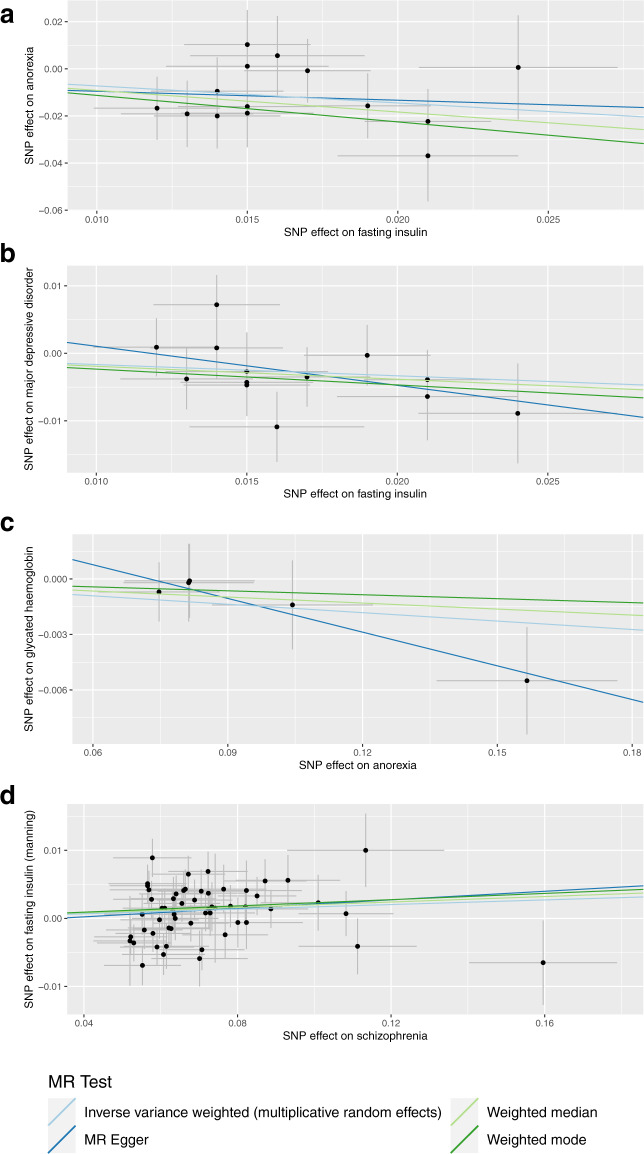
Sensitivity analyses of causal estimates. The scatterplots represent the IV effects on the exposure and outcome variables (black point), with the confidence intervals for both estimates denoted by the horizontal and vertical lines, respectively. Each coloured slope is indicative of the causal effect of a unit increase in the exposure on the outcome, estimated by the method in the legend utilised to shade the trendline – that is, inverse-variance weighted effect with multiplicative random effects (light blue), weighted median (light green), weighted mode (dark green), and MR-Egger (dark blue). The four panels correspond to a different exposure-outcome pair: (**a**) fasting insulin → anorexia nervosa, (**b**) fasting insulin → major depressive disorder, (**c**) anorexia nervosa → HbA1c, and (**d**) schizophrenia → fasting insulin.

We subjected the causal estimate of fasting insulin on the risk for anorexia to a suite of sensitivity analyses to assess the rigor of our derived IVW estimate and evidence of violations of core MR assumptions (Fig. 2a, Supplementary Tables 3–6). No IVs were detected as outliers using the MR-PRESSO approach. Furthermore, the weighted median model supported the putative protective effect of elevated fasting insulin on risk for anorexia derived from the IVW (OR_Weighted Median_ = 0.40, [95% CI:0.21–0.77], *P* = 6.3 × 10^−3^), as did the weighted mode estimator (OR_Weighted Mode_ = 0.32 [95% CI: 0.13–0.84], *P* = 0.037). The MR-Egger model was not significant; however, the causal estimate was in the same direction of effect as the other two approaches, albeit with an extremely wide confidence interval (OR_Egger_ = 0.68, 95% CI: 0.05–9.10). It should be noted that the MR-Egger method typically has notably less power than other approaches, particularly when fewer IVs are used [35]. The causal estimate between fasting insulin and anorexia using both the MR-PRESSO and GSMR approaches was also practically identical to the IVW estimator, although the GSMR estimate displayed a larger standard error (OR_GSMR_ = 0.48, [95% CI:0.30–0.79], *P* = 3.7 × 10^−3^). There was no compelling evidence for unbalanced pleiotropy amongst IVs utilised in this construct: heterogeneity between IV effects was not significant (*Q* = 8.24, *df* = 13, *P* = 0.827), the intercept of the MR egger regression did not significantly differ from zero (intercept = −5.7 × 10^−3^, *P* = 0.795), and the MR PRESSO test of global pleiotropy was also not significant. A leave-one out recalculation of the causal estimate did not indicate that a single IV or subset of IVs were unduly influencing the model.

### The impact of BMI adjustment on fasting insulin instrumental variables

We observed a more significant effect of fasting insulin on AN liability using fasting insulin IVs from a GWAS in which covariation for BMI was applied. Given the putative bidirectional relationship between anorexia and BMI [46], we sought to investigate evidence of BMI related confounding on our causal estimate. First, the variance explained by the insulin IVs adjusted for BMI was larger (0.69%) than the unadjusted IVs (0.52%), suggesting greater power to detect an effect using the BMI adjusted SNPs. We directly compared the effect size of the BMI adjusted IVs in the insulin GWAS to the same SNPs without BMI adjustment to identify stable fasting insulin IVs which reached genome-wide significance regardless of covariation for BMI (Fig. 1b). There were eight such IVs which were significantly associated with fasting insulin irrespective of BMI adjustment (Supplementary table 7). The causal estimate of fasting insulin on AN was recalculated using only these eight IVs, with both the BMI adjusted and unadjusted IV-insulin effect sizes yielding a significant protective impact of fasting insulin on liability to AN [IVW with multiplicative random effects estimate]—BMI adjusted stable IVs: OR = 0.55 [95% CI: 0.32–0.95], *P* = 0.03; BMI unadjusted stable IVs: OR = 0.55 [95% CI: 0.32–0.93], *P* = 0.027 (Fig. 1c). The change in IV-fasting insulin effect size upon covariation for BMI was relatively small in most instances, with no reversal of the direction of effect relative to the tested allele (Supplementary Table 7). Importantly, this stable subset of IVs remained sufficiently strong, as quantified by the *F*-statistic: *F*_Adjusted BMI_ = 53.33, *F*_Unadjusted_ = 38.09. These data support that the utilisation of BMI adjusted IVs does not constitute collider bias, although this possibility cannot be definitively excluded. Moreover, we identified five fasting insulin IVs which were also associated with BMI at genome wide significance (*P* < 5 × 10^−8^) and recalculated the IVW estimate with these instruments removed. The effect size observed in this reduced IVW model was not greatly attenuated (OR = 0.51 [95% CI: 0.32–0.83], *P* = 7.03 × 10^−3^, Supplementary Table 8), supporting that the relationship between insulin and anorexia was not unduly biased by horizontal pleiotropy through IV effects on BMI.

### Psychiatric disorders as the exposure phenotype

Bidirectional relationships were investigated using a reverse MR approach, whereby the psychiatric disorders were the exposures and the glycaemic traits acting as outcomes. There were five disorders with at least three IVs which overlapped the outcome GWAS (ADHD, AN, BP, MDD, and SZ), and thus, these phenotypes were considered as exposures. We demonstrated weak evidence of a positive causal effect of genetic liability to SZ on fasting insulin (*β* = 0.017 [95% CI: 0.004–0.03], *P* = 0.016) and a negative relationship between AN and HbA1c (*β* = −0.015 [95% CI: −0.03 to −0.002], *P* = 0.023), although these estimates did not survive multiple testing correction. Causal estimates using binary exposures are difficult to intuitively interpret given a unit increase represents a 2.72-fold multiplicative increase in the odds of the disorder [72]. Previously, it has been suggested that these estimates could be converted to the liability scale to represent the change in the outcome per standard deviation increase in liability to the disorder [53]. The converted IVW results assumed a 0.7% and 0.9% population prevalence of SZ and AN, respectively: *β* = 0.048 (SZ → fasting insulin), *β* = −0.041 (AN → HbA1c). We caution that binary exposures must be carefully interpreted in MR analyses, and thus, these models may be more appropriately considered as a test of the null hypothesis rather than a direct estimation of effect size, as discussed elsewhere [72]. The IVs selected for these two psychiatric exposures explained 0.26% (*F* = 37.73) and 2.29% (*F* = 43.37) of the phenotypic variance for AN and schizophrenia, respectively. There were no significant estimates between any of the remaining psychiatric exposures and glycaemic traits as outcomes (Supplementary Table 2*)*. The IVW estimates for all exposure-outcome pairs, in both MR directions, are detailed in Table 3.

**Table 3:** Causal relationships between glycaemic traits and psychiatric disorders estimated via two-sample Mendelian randomisation using an inverse-variance weighted effect model with multiplicative random effects.

Trait one	Trait two	Glycaemic → psychiatric (beta)^a^	Psychiatric → glycaemic (beta)^b^
ADHD	Fasting insulin	0.21 (0.34)	−0.0004 (0.02)
ADHD	Fasting glucose	0.20 (0.12)	−0.008 (0.02)
ADHD	HbA1c (all)	0.34 (0.20)	−0.02 (0.01)
ADHD	HbA1c (glycaemic)	0.45 (0.37)	N/A
AN	Fasting insulin	−0.72 (0.20)^**^	−0.0005 (0.02)
AN	Fasting glucose	−0.12 (0.13)	−0.003 (0.03)
AN	HbA1c (all)	0.05 (0.21)	−0.02 (0.01)^*^
AN	HbA1c (glycaemic)	−0.28 (0.36)	N/A
ASD	Fasting insulin	−0.06 (0.30)	N/A
ASD	Fasting glucose	−0.14 (0.12)	N/A
ASD	HbA1c (all)	−0.16 (0.15)	N/A
ASD	HbA1c (glycaemic)	−0.38 (0.26)	N/A
BP	Fasting insulin	−0.20 (0.52)	0.003 (0.01)
BP	Fasting glucose	−0.15 (0.18)	−0.009 (0.01)
BP	HbA1c (all)	−0.10 (0.15)	0.012 (0.01)
BP	HbA1c (glycaemic)	−0.51 (0.46)	N/A
MDD	Fasting insulin	−0.17 (0.07)^*^	−0.01 (0.02)
MDD	Fasting glucose	0.02 (0.04)	0.02 (0.02)
MDD	HbA1c (all)	0.09 (0.05)	0.01 (0.02)
MDD	HbA1c (glycaemic)	−0.11 (0.13)	N/A
OCD	Fasting insulin	0.14 (0.65)	N/A
OCD	Fasting glucose	−0.19 (0.23)	N/A
OCD	HbA1c (all)	0.40 (0.43)	N/A
OCD	HbA1c (glycaemic)	0.38 (0.54)	N/A
SZ	Fasting insulin	0.19 (0.29)	0.02 (0.01)^*^
SZ	Fasting glucose	−0.13 (0.10)	0.008 (0.01)
SZ	HbA1c (all)	0.01 (0.14)	−0.0005 (0.004)
SZ	HbA1c (glycaemic)	−0.36 (0.26)	N/A
TS	Fasting insulin	0.42 (0.42)	N/A
TS	Fasting glucose	0.15 (0.19)	N/A
TS	HbA1c (all)	0.16 (0.33)	N/A
TS	HbA1c (glycaemic)	0.98 (0.56)	N/A

Mendelian randomisation (IVW estimator with multiplicative random effects) was performed in both directions (subject to the availability of IVs), that is, glycaemic traits as the exposure (glycaemic **→** psychiatric), along with psychiatric disorders as the exposure. The glycaemic traits were as follows: fasting insulin (BMI adjusted, BMI unadjusted estimates available in Supplementary Tables 1 and 2), fasting glucose, glycaeted haemoglobin (all IVs = HbA1c (all), IVs annotated as glycaemic = HbA1c (glycaemic). Bolded beta estimates are statistically significant. N/A represents analyses where less than three overlapping IVs were available.

The psychiatric traits were: *ADHD* attention deficit hyperactivity disorder, *AN* anorexia nervosa, *ASD* autism spectrum disorder, *BP* bipolar disorder, *MDD* major depressive disorder, *OCD* obsessive compulsive disorder, *SZ* schizophrenia, *TS* Tourette’s syndrome.

^a^IVW beta estimates (standard error) of the effect of glycaemic on the risk of psychiatric disorders represent the log odds of the disorder per unit increase of the exposure. The unit of effects were as follows: fasting insulin = natural log transformed pmol/L, fasting glucose = mmol/L, HbA1c = % HbA1c.

^b^IVW beta estimates (standard error) using the psychiatric disorders as exposures represent the effect on glycaemic traits per 2.72-fold multiplicative increase in the odds of the psychiatric disorder, however, we treat this primarily as a test of the null hypothesis.

*P < 0.05, **P < 0.01.

We applied a subset of these sensitivity analyses (MR Egger, Weighted Median, and Weighted Mode estimator) to the three remaining IVW estimates which did not survive multiple testing correction (uncorrected *P* < 0.05) to evaluate the consistency of the causal estimate (Fig. 2b–d). All three exposure-outcome pairs had consistent point estimate effect directions, that is, a protective effect of fasting insulin on the risk of MDD (negative beta), a positive causal estimate between SZ and fasting insulin (positive beta), and a negative estimate between AN and HbA1c (negative beta). However, these models were only statistically significant in the case of the weighted median estimator of the schizophrenia to fasting insulin construct (Supplementary Table 3), with the confidence interval overlapping the null in most instances. As a result, we classified these trait pairs as having relatively weak evidence of causation in comparison to the fasting insulin to anorexia model.

### Assessment of the direction of causal effect between fasting insulin and anorexia nervosa

The MR models implemented in this study assume that the IVs impact the exposure, which in turn affects the outcome—however, in practice it is feasible that the orientation of the causal pathway is incorrect and that the outcome influences the exposure through the genetic instruments. To address this, we performed a Steiger directionality test. This method examined whether the phenotypic variance in the outcome explained by the IVs is less than that of the exposure to test the assumed causal direction. We calculated the variance in anorexia risk explained by the IVs and converted to the liability scale using the upper (4%) and lower (0.9%) bound of estimated population prevalence for anorexia [28]. The Steiger directionality test supported the hypothesis that the effect of fasting insulin on risk for anorexia is the correct causal direction as the variance explained by the IVs was lower for anorexia than fasting insulin (*P* = 1.35 × 10^−27^, *P* = 1.36 × 10^−27^ respectively for the upper and lower bound anorexia prevalence estimates) (Supplementary Table 9). Furthermore, the reverse IVW estimator did not indicate evidence of an effect from AN to fasting insulin (*P* = 0.986), although there was weak evidence of a negative relationship between anorexia liability and HbA1c, as described in the previous sections. Given the genetic correlation between fasting insulin and anorexia, we used a latent causal variable model to determine the proportion of trait one (fasting insulin) that genetically causes trait two (anorexia), which was quantified as the mean posterior estimate of the GCP. The sign of the mean posterior GCP estimate suggests that fasting insulin is partially genetically causal for anorexia; however, this was not significantly different from zero, likely due to the large standard error ($\widehat {GCP}$ = 0.39, *SE* = 0.33, *P* = 0.26]. Whilst the LCV GCP estimate was not significant, there was strong evidence that the causal direction was not anorexia to insulin [H_0_: GCP= −1, *P* = 3.6 × 10^−104^], in accordance with the Steiger directionality test results (Supplementary Table 10).

## Discussion

Glycaemic regulation is involved with many physiological processes, however, its role in neurological function has motivated investigation of this system in psychiatric disorders. Using a MR approach which leverages genetic IVs as proxies for three glycaemic traits, we uncovered evidence of a protective effect of elevated fasting insulin on the risk for anorexia nervosa. No strong evidence of a causal effect of any of the glycaemic traits was found testing seven other psychiatric phenotypes, although there was relatively weaker support for a protective effect of fasting insulin on depression. Notably, we did not replicate a previous study which demonstrated a risk increasing effect of fasting insulin on schizophrenia, however, we utilised a larger schizophrenia GWAS and different IVs [73]. Although previous analysis has shown a relationship between first episode psychosis and glycaemic dysregulation, and elevated rates of dysglycaemia in psychiatry, this was not supported by our MR model. Our inability to detect this relationship may be limited by the strength of the IV used, or the observed effects from previous analysis may be due to variables with shared genetic liability which influence glycaemic homoeostasis, such as inflammation or BMI. Interestingly, this study demonstrated there was weak evidence of a causal effect in the opposite direction, whereby genetic liability to schizophrenia was associated with increased fasting insulin, supporting previous data which demonstrated an association between schizophrenia PRS and insulin resistance [29]. We prefer to treat these binary exposure estimates as a test of the null hypothesis as the interpretation of effect sizes from binary exposures are not intuitive and may be subject to unrealistic assumptions related to the homogeneity of their effects [72]. The future availability of more data with the power to explain a larger portion of the variance in the exposures and outcomes, could yet yield more evidence of a causal relationship between dysglycaemia and other psychiatric disorders. The negative relationship between elevated insulin and anorexia risk derived in this study supports the negative genetic correlation observed between the two GWAS studies by LDSC [28]. However, it should be noted that genetic correlation between traits may confound MR estimates, which cannot be definitively ruled out in this study as our LCV estimate of partial genetic causality was not significantly different than zero. The association between a natural log transformed pmol/L increase in fasting insulin and odds of anorexia yielded an odds ratio of 0.48 [95% CI: 0.33–0.71]. To contextualize this unit of effect, we considered fasting insulin values from a large cohort of 10.5–11 year old female normal weight European participants [74]. We estimate that a unit increase from the 10th percentile of this cohort (with a fasting insulin concentration of 30.97 pmol/L) would correspond to ~84.19 pmol/L, which is roughly equivalent to the 90th percentile of the cohort (~86.91). This estimate derived from the IVW model was supported by sensitivity analyses which did not indicate any statistical evidence of unbalanced pleiotropy which would confound the IVs we selected.

The role of insulin signalling and glucose metabolism in the brain, and its interplay with the periphery, is complex, necessitating further research to specifically understand how fasting insulin could exert a protective effect on anorexia. The relationship between circulating insulin and weight gain may contribute to this protective effect given peripheral insulin and insulin therapy in the context of diabetes is associated with weight gain [42, 75]. There are a number of mechanisms by which this is proposed to be mediated, including the stimulatory effect of insulin on fatty acid storage and cell growth and a reduction in glycosuria [76–78]. Furthermore, MR analyses have supported a positive relationship between insulin and weight gain [79, 80]. Given the nature of the clinical presentation of anorexia, the effect of insulin on hunger and satiety is particularly pertinent. Increased insulin levels in the body results in higher levels of hunger and an increased pleasantness associated with sweet taste [81]. This corresponds to data from anorexia cohorts which report that individuals with anorexia have a reduced appreciation of sweet tastes [82]. In contrast, insulin is postulated to have an anorexigenic effect in the brain [83, 84], partly through its inhibition of the orexigenic agouti-related peptide and neuropeptide Y neurons [85, 86]. This may contradict the putative risk-decreasing effect of insulin on anorexia we observed in our study, however, there is evidence of a significant sexual dimorphism in this phenomenon. An example of this has been demonstrated using intranasal insulin administration, in which hunger was decreased only in male participants, conversely, there were positive cognitive enhancing effects seen only in women [87], although this was a small study (*N* = 32) that warrants replication in a larger cohort. This sexual dimorphism in the effect of insulin signalling is further supported by rodent data [88]. As anorexia is significantly more prevalent in females [89] it is possible that the sexual dimorphic effect of insulin on hunger signalling is contributing to this discrepancy in prevalence. However, the data supporting the central nervous system impact of insulin in humans are derived from studies with small sample sizes, and thus, further work is needed to resolve the relationship between insulin signalling and satiety. Moreover, many insights into the neurological consequences of insulin signalling arise from rodent models and caution must be exercised when directly extrapolating physiology from these models to humans.

There are a number of future directions which arise from these data. Only Europeans were used in this analysis warranting its extension to trans-ethnic cohorts. As the negative relationship between insulin and anorexia is also evidenced using a genomic correlation approach, there is a need to investigate shared genes and biological pathways which may explain this association. This would be particularly valuable to interpret the causal estimate we uncovered, as individuals who develop anorexia may be genetically predisposed to have altered glycaemic homoeostasis. Furthermore, a well-powered, sex stratified GWAS of anorexia could be utilised to formally test whether the impact of insulin on anorexia risk displays sexual dimorphism, with sexual dimorphisms likely also evident in glycaemic traits themselves. As diagnosis of anorexia is highly skewed towards females, it will likely be a continued challenge to genotype larger male cohorts which approach the sample sizes available for female participants. There are also a number of other heritable psychiatric phenotypes for which the effect of glycaemic traits could be tested using MR, including measures like neuroticism and anxiety. It is also important to consider the inherent limitations of MR in light of our data. We did not uncover any statistical evidence of unbalanced pleiotropy amongst the fasting insulin IVs; however, this cannot be definitively proven and future replication in larger studies is paramount. Moreover, whilst the IVs selected for insulin were appropriately strong as quantified by an *F*-statistic, they still only explain a fraction of the phenotypic variance in fasting insulin. Despite these caveats and other methodological challenges associated with causal inference using IVs, we believe the fasting insulin—anorexia model to be reliable. In conclusion, we uncovered evidence of a protective effect of fasting insulin on the risk of anorexia nervosa, with further work now required to further understand the biological mechanisms underpinning this relationship.

## Funding and disclosure

MJC is supported by a National health and medical research council (NHMRC) Senior Research Fellowship (1121474) and an NHMRC project grant (1147644), URL: https://www.nhmrc.gov.au/. The funders had no role in study design, data collection and analysis, decision to publish, or preparation of the manuscript. The authors declare no competing financial interests.

## Supplementary information

Related Manuscript File
